# A Comparative Study of CO_2_ Forecasting Strategies in School Classrooms: A Step Toward Improving Indoor Air Quality

**DOI:** 10.3390/s25072173

**Published:** 2025-03-29

**Authors:** Peio Garcia-Pinilla, Aranzazu Jurio, Daniel Paternain

**Affiliations:** 1 Institute of Smart Cities (ISC), Public University of Navarra (UPNA), Campus de Arrosadia, 31006 Pamplona, Spain; peio.garcia@unavarra.es (P.G.-P.); aranzazu.jurio@unavarra.es (A.J.); 2 inBiot Monitoring, PºSantxiki, 2 LB5, 31192 Mutilva Alta, Spain

**Keywords:** air quality sensors, indoor air quality, forecasting, pollutants, machine learning, air quality modeling

## Abstract

This paper comprehensively investigates the performance of various strategies for predicting CO_2_ levels in school classrooms over different time horizons by using data collected through IoT devices. We gathered Indoor Air Quality (IAQ) data from fifteen schools in Navarra, Spain between 10 January and 3 April 2022, with measurements taken at 10-min intervals. Three prediction strategies divided into seven models were trained on the data and compared using statistical tests. The study confirms that simple methodologies are effective for short-term predictions, while Machine Learning (ML)-based models perform better over longer prediction horizons. Furthermore, this study demonstrates the feasibility of using low-cost devices combined with ML models for forecasting, which can help to improve IAQ in sensitive environments such as schools.

## 1. Introduction

In the shadows of the SARS-CoV-2 pandemic, studying Indoor Air Quality (IAQ) remains a major global need. It is well known that people spend at least 90% of their time indoors [1] and that exposure to poor IAQ is responsible for the deaths of 3.8 million people annually [2]. The demand for IAQ solutions has grown significantly in recent years, with an urgent need to pay more attention to educational buildings and other public spaces in general [3,4]. In fact, the World Health Organization (WHO) emphasizes the need to study monitoring tools and new technologies to protect health in this area [5]. In particular, carbon dioxide (CO_2_) plays a key role in IAQ, negatively affecting the concentration and productivity of people in indoor spaces [6].

Schools are particularly important environments due to the extensive time children spend in classrooms during their formative years. Students typically accumulate 15,600 h in the classroom by the end of high school, exceeding the time they spend anywhere else besides home [7]. Children are more sensitive to the adverse health effects of indoor pollution, as their higher breath-to-body weight ratio (50% higher than adults) causes them to inhale more air. Consequently, higher indoor air pollution exposes them to more pollutants, including CO_2_, which increases their vulnerability to the associated risks [8]. Furthermore, several studies in school environments have found an association between elevated levels of CO_2_ in classrooms and neurophysiological symptoms such as headaches and fatigue [9,10]. Notably, a 30% reduction in academic performance has been observed in classrooms with high CO_2_ levels, while improved ventilation and thermal comfort enhance productivity [11,12,13,14].

Classrooms often rely on natural ventilation (i.e., opening windows), the effectiveness of which is conditioned by human intervention, as individuals must decide when and how to open them. This subjective and imprecise management can be problematic, especially during colder months or in regions with strict window-opening policies. As a result, classrooms are more vulnerable to CO_2_ accumulation, particularly when they are fully occupied for extended periods. Although solutions such as window-opening detection systems or intelligent ventilation mechanisms could mitigate this issue, their implementation involves high costs and the need to modify school infrastructure, which is not always feasible. In many cases, public schools operate with limited budgets that hinder large structural works. Therefore, it is important to find solutions that avoid altering the school infrastructure while effectively reducing pollution, particularly CO_2_ levels.

In this context, monitoring technologies based on Internet of Things (IoT) devices offer a promising solution. These devices provide real-time environmental data, enabling more informed decision-making during periods of high pollution [15]. While IoT tools are valuable for immediate response, they do not inherently provide a means of controlling future pollution levels. This is where forecasting models can play a key role.

Unlike real-time monitoring, forecasting focuses on predicting future pollutant behavior based on historical data. By anticipating CO_2_ levels in classrooms, early decisions can be made to avoid direct exposure to unhealthy conditions. In this way, forecasting models can provide an economical and effective solution, particularly in classrooms, where natural ventilation patterns are difficult to predict. Instead of detecting when a window is physically open, patterns in the data can be learned and used to predict future CO_2_ levels. Consequently, forecasting techniques for estimating CO_2_ and other pollutants in IAQ have gained significant importance in recent literature.

Historically, statistical techniques have dominated the approach to forecasting problems in Time Series (TS), with notable examples including Auto-Regressive Integrated Moving Average (ARIMA) [16]. In contrast, since 2015 there has been a significant increase in the number of published research using Machine Learning (ML) to address IAQ-related challenges [17]. This trend is also evident in educational settings, where several studies have demonstrated the effectiveness of ML models in predicting indoor pollutants.

For instance, ref. [18] explored different architectures of simple Artificial Neural Networks (ANN) trained using simulator-generated data to predict both energy consumption and pollutant concentrations in school classrooms. Similarly, ref. [19] collected measurements of CO_2_, PM2.5 (particulate matter measuring 2.5 microns or less), and VOCs (volatile organic compounds) at a daycare center in May 2021. Their study compared the performance of various ANN-based architectures for 5-min pollutant prediction. Additionally, research has investigated models using Recurrent Neural Network (RNN) architectures to predict 6-h pollution levels, aiming to reduce asthma attacks in children [20]. In [21], the authors deployed fifty IoT-based sensors to intermittently collect IAQ data from different classrooms in a Taiwanese school during the period from September 2016 to December 2018. Due to the inherent collinearity of the data, decision tree-based techniques, specifically XGBoost and Random Forest (RF), were chosen to predict PM2.5 values. Another study by [8] gathered IAQ data using low-cost sensors installed in ten classrooms at a school in northern China between March 2018 and February 2019. This research adopted a different approach, focusing on predicting PM2.5 levels by employing genetic algorithms to generate a specific model for each month of the year. In other research, ML techniques have been applied to solve school IAQ-related challenges that do not involve forecasting. These studies have addressed broader issues such as assessing health impacts related to asthma and allergies [22,23] as well as predicting comfort indicators such as productivity and thermal comfort [14].

In recent years, the rise of ML techniques has introduced new possibilities for TS forecasting, ranging from traditional models such as Random Forest [24] and XGBoost [25] to innovative Deep Learning (DL) architectures such as N-HiTS [26] and Temporal Convolutional Networks (TCNs) [27]. Convolutional Neural Networks (CNNs) and their extensions, including TCNs, have emerged as promising alternatives to RNNs for TS forecasting. While RNNs, particularly Long Short-Term Memory networks (LSTMs) and Gated Recurrent Units (GRUs), have been the preferred choice for the past two decades thanks to their ability to effectively model sequential dependencies [28,29], recent studies have shown that CNN-based architectures such as TCNs can achieve comparable or even superior performance in terms of predictive accuracy and computational efficiency [30,31,32]. This shift highlights the growing potential of CNN-based models to advance forecasting methodologies.

Despite advances in pollutant forecasting and growing evidence supporting the potential of ML techniques, several persistent challenges limit the robustness and generalizability of existing models in real-world scenarios:

- Use of Real-World Datasets: Many studies rely on publicly available or simulated datasets, which may not accurately reflect real-world conditions, thereby introducing noise and potentially biasing models.
- Validation Techniques: Inadequate validation strategies can affect model performance and generalization. Many studies provide limited details on the validation methods used or apply traditional ML techniques that do not account for temporal dependencies in the TS data. Adopting specialized TS validation techniques would provide more robust insights.
- Prediction Horizons: Although model performance varies across different prediction horizons, systematic comparisons are rare. In IAQ forecasting, earlier predictions allow more time for corrective actions such as activating ventilation systems. Without systematic comparisons, it is difficult to understand how the effectiveness of the model changes over time and how timing impacts decision-making.
- Comparison with Simple Models: Complex DL architectures are often assumed to outperform simpler models. However, in short-term predictions, simpler models with lower computational costs can achieve similar accuracy. Whether or not complex ML models consistently outperform simpler methods in TS forecasting remains an open question [33,34].
- Statistical Techniques for Reliability: Statistical methods that enhance the reliability and validity of forecasting results are underutilized in the literature. The absence of rigorous statistical validation can undermine the credibility of model performance and its generalization to real-world scenarios.
- Scale-Independent Performance Metrics: Many studies rely on traditional ML performance metrics that are scale-dependent, which complicates the comparison of forecasting models across different TS datasets. The use of scale-independent metrics is essential for more reliable and comparable evaluations.

Based on these identified gaps, it is evident that a more rigorous and comprehensive approach is needed to advance CO_2_ forecasting methodologies in schools. In this paper, we address these challenges by proposing a novel study designed to evaluate different forecasting strategies across multiple prediction horizons. By exploring a variety of methodologies and temporal settings, this research not only highlights the most effective approach for CO_2_ forecasting in classrooms but also contributes to a better understanding of how these strategies perform under various temporal conditions.

To achieve this objective and effectively address the identified challenges, this study is designed with the following settings:

- Real-World Data Collection: CO_2_ levels were continuously monitored in fifteen schools located in Navarra, Spain using IoT devices from 10 January 2022 to 3 April 2022.
- Forecasting Methodologies: Three distinct strategies were implemented: simplistic methods (Shifted Model and Moving Average Model), a statistical approach (ARIMA), and ML-based models (XGBoost, Random Forest, N-HiTS, and TCN), allowing for a comprehensive evaluation of forecasting effectiveness and complexity.
- Prediction Horizons: The forecasting models were tested across six different horizons ranging from 10 min to 4 h in order to systematically assess how model performance varies over different temporal windows.
- Validation and Statistical Testing: A rolling cross-validation scheme was employed to account for temporal dependencies, ensuring robust and unbiased performance evaluation. Additionally, statistical tests were conducted to validate the results, enhancing the reliability and credibility of the findings. Scale-independent performance metrics were used to enable consistent and fair comparisons across models.

This study not only provides a tailored solution for CO_2_ forecasting in schools but also establishes a rigorous methodological framework that can guide future research in IAQ forecasting. By addressing the existing gaps and implementing a holistic approach, this research contributes to the advancement of ML-based forecasting methodologies, fostering more robust, generalizable, and reliable IAQ models. Moreover, the findings are expected to offer practical insights to support decision-making in educational institutions, ultimately enhancing IAQ management.

It should be noted that the devices used in this study are low-cost IAQ monitors and do not track external factors such as window opening, which as explained previously can influence CO_2_ levels and are also difficult to control. The proposed methodology is not presented as a definitive solution to reduce CO_2_ pollution in classrooms but rather as a cost-effective and practical approach. Recognizing that ventilation in classrooms is poor and irregular, it attempts to generalize the problem and control external variables without requiring any structural modifications to classrooms.

The structure of this paper is as follows: Section 2 details the experimental process, from data collection to the entire methodology employed, the results and discussion of the models are presented in Section 3, and conclusions are provided in Section 4.

## 2. Materials and Methods

**Figure 1:**
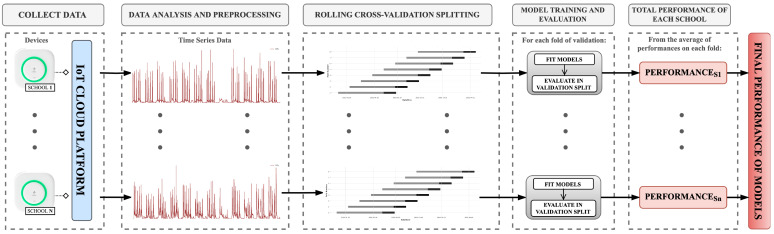
General workflow of the steps conducted during the experiment.

Recalling that the main objective of this paper is to conduct a comprehensive study evaluating the performance of several forecasting architectures from the literature on the specific problem of predicting CO_2_ in classrooms using IoT sensors considering different prediction horizons, Figure 1 presents the flowchart of the methodology of the study. The flowchart is separated into five different steps: (1) data collection, (2) data analysis and preprocessing, (3) data partitioning for validation, (4) model training, and (5) model evaluation. The following subsections describe each step of the experiment in detail.

### 2.1. Data Collection

#### 2.1.1. Study Area

The study was conducted in fifteen schools (denoted schools $S_{1},\ldots ,S_{15}$) located in the metropolitan area of Pamplona, the main city of Navarra, Spain (42°49′ N, 1°39′ W). Data were collected during the school period from 10 January 2022 to 3 April 2022. Each device was installed inside a selected classroom in each school to continuously monitor CO_2_ levels.

#### 2.1.2. Devices

**Figure 2:**
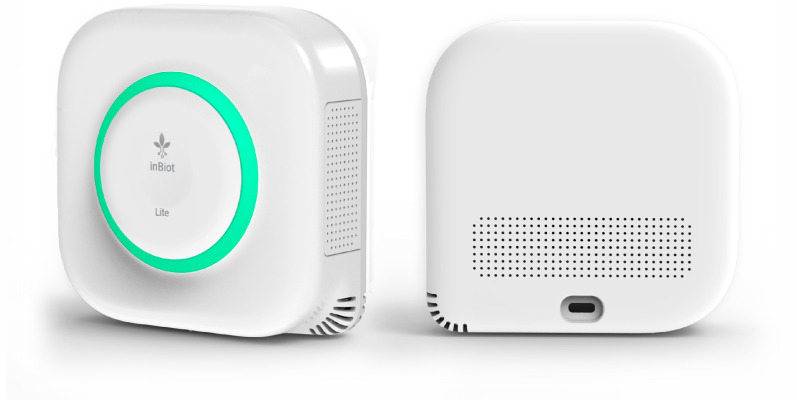
The MICA device used to measure the concentration of CO_2_ in classrooms. Proprietary design by Inbiot Monitoring SL (www.inbiot.es, accessed on 23 January 2025).

The data for this study were collected using IoT-based IAQ monitoring devices called MICAs. MICA devices are manufactured and commercialized by the IAQ specialist company InBiot Monitoring SL (www.inbiot.es, accessed on 23 January 2025). Figure 2 shows the design of the device.

These devices are equipped with low-cost sensors that enable real-time measurement of various indoor pollutants, including CO_2_. The CO_2_ sensor uses NDIR technology and measures concentration in parts per million (ppm). The measurement range is 0–5000 ppm and the accuracy offered by the sensor is ±(50+3%m.v.) ppm. The sensor records measurements every 10 min. Before installation in the classrooms, the devices were tested and calibrated in the laboratory according to established quality control procedures in order to ensure proper sensor operation and regular data transmission. After installation, the sensors underwent a preheating period during which they autonomously adjusted their measurements according to the reference concentration of the space for the first 24 h. The MICA device is certified by RESET accreditation [35], satisfying the established criteria for precision and resolution.

### 2.2. Data Analysis and Preprocessing

Following the monitoring period from January to April 2022 (academic year 2021/2022), a total of 181,440 CO_2_ measurements were collected, with 12,096 data points for each school. Table 1 summarizes the basic statistics of the CO_2_ measurements for each school, distinguishing the measurements into occupied and non-occupied hours in order to analyze the impact of classroom occupancy. Occupied hours were defined as weekdays from 8:00 am to 5:00 pm, excluding weekends and vacation periods. The results show that the mean global CO_2_ concentrations were close to the baseline level of 400 ppm (448.25 ppm). This indicates that the classrooms were predominantly in non-occupied condition during the monitoring period. However, during occupied hours the mean values were higher (452.57 ppm) compared to non-occupied periods (406.68 ppm), reflecting the impact of human presence as the primary CO_2_ source. Additionally, the maximum values recorded during occupied hours were notably higher, frequently exceeding 1000 ppm in several schools, while non-occupied periods showed no significant peaks.

**Figure 3:**
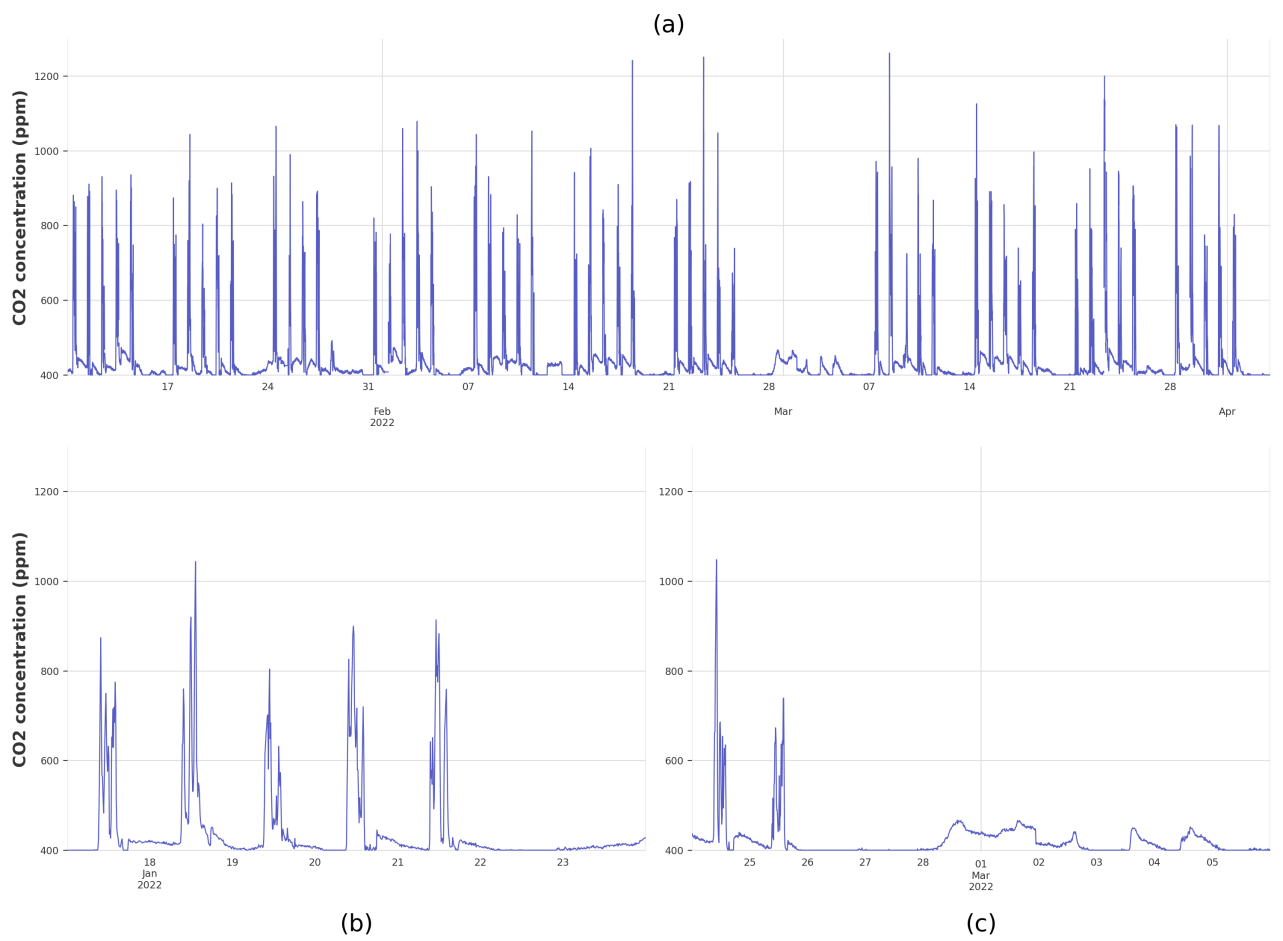
Evolution of the CO_2_ concentration (ppm) in School S_3_. The top subfigure (**a**) plots all data for the entire study period, while subfigure (**b**) shows the evolution in a typical week of a classroom with occupancy and subfigure (**c**) shows a week with almost no occupancy.

Figure 3 shows an example of the trend of the variable measured in School S_3_, while Figure 3a plots the evolution of CO_2_ over the entire study period (January 2022–April 2022). Weekly patterns are evident in the data, reflecting the typical school occupancy schedule. Figure 3b provides a closer view of specific week, where the evolution of CO_2_ can be seen on weekdays (Monday to Friday) along with the fact that no occupancy occurs during the evenings, parts of the afternoons, or weekends. However, it is important to note that not all weeks are exactly the same, as there are bank holidays and other periods of lower activity with almost no occupancy, such as the carnival holidays (between 27 February 2022 and 7 March 2022), as shown in Figure 3c. Carnival festivals are not official holidays, and it is the responsibility of each school to determine their specific dates, which consequently vary from one school to another.

#### Preprocessing

To improve the predictive capacity of the studied models, data preprocessing was necessary to enhance their quality. The use of low-cost IoT sensors may cause challenges such as missing records. In this study, missing values were imputed using linear interpolation, resulting in a total of 8923 imputed values, which accounted for 4.92% of the entire dataset. For the application of some of the models discussed below, normalization of the values to the interval [0,1] was performed.

### 2.3. Data Partitioning for Validation

In TS forecasting, separating the data into distinct training and validation sets is crucial to prevent bias and ensure model generalizability. In ML, it is common to split the dataset solely into training and validation sets, leaving the last portion of the data for validation. However, in our specific scenario this approach may not accurately reflect reality, as the behavior of the pollutant varies across weeks.

In this study, we employed a Rolling Cross Validation (Rolling-CV) technique, which is typically used for hyperparameter optimization (fine-tuning). However, we also applied it to validate model performance over different time periods. The measurements from each school were divided into eight different subsets (*Folds*). In each fold, 80% of the data (4 weeks) were used for training and 20% (1 week) were used for validation. We ensured that each validation set covered at least one full week of data. In this case, a variant of Rolling-CV with overlap was used, where the validation data were included as training examples for the next set, with an offset of one week between folds. Table 2 shows the temporal distribution of the folds over time.

**Figure 4:**
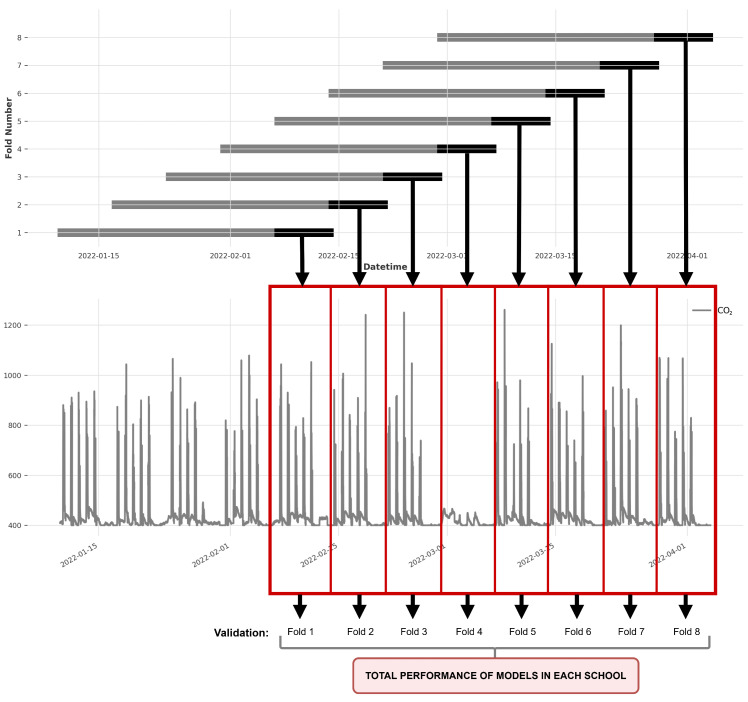
Representation of Rolling-CV partitioning on the complete dataset of School S_3_.

Figure 4 provides a visual representation summarizing the Rolling-CV structure applied to the data from School S_3_. This segmentation approach generates diverse validation sets, capturing various patterns across different time periods. Notice how the validation period corresponding to Fold 4 refers to the carnival holiday week mentioned in Section 2.2, which is characterized by minimal classroom occupancy.

### 2.4. Model Training

We evaluated seven forecasting models encompassing three primary methodological approaches commonly used for TS problems.

First, a block of two simple models requiring minimal computational resources and no prior learning served as a baseline for comparison. The Shifted (SH) model shifts the TS by a constant value, providing a naïve method for predicting TS behavior in scenarios with consistent trends. The Moving Average (MA) model uses the average of the most recent *N* data points for prediction, effectively smoothing out noise and short-term fluctuations.

The second approach was statistical, employing the widely adopted ARIMA model [16], which is commonly used in TS forecasting. ARIMA was selected due to its ability to model linear relationships in TS data while handling both stationary and nonstationary components.

Finally, the third approach reflected the growing use of ML techniques. Random Forest (RF) and XGBoost (XGB) were included as representative classical ML models. These models were chosen for their ability to handle complex nonlinear relationships in data, with RF being effective at reducing overfitting through ensemble learning and XGBoost offering strong performance on high-dimensional data. Additionally, two architectures for DL models were included. The first, N-HiTS [26], is based on the recent N-BEATS [36] architecture, which has demonstrated promising results in long-horizon TS forecasting by efficiently capturing both trend and seasonality components. On the other hand, TCN was included due to its ability to efficiently capture long-range dependencies via dilated convolutions [27], support parallelization for faster processing of long sequences [37], and avoid the vanishing gradient issues found in RNNs [38].

The selection of these models aimed to cover a broad range of methodological approaches, from simple baseline models to statistical and ML techniques, thereby ensuring a comprehensive comparison of their performance across different TS forecasting scenarios.

Regarding the specific parameters of each model, in SH the number of shifts was defined by the prediction horizon. For MA, a data frame equivalent to six records (1 h) was established as the window size. The optimal parameters of the ARIMA model were estimated using the AutoARIMA implementation offered by the DARTS [39] library. In the case of RF, 100 trees were used in the forest, which were grown until all leaves had been generated. In both the RF and XGB models, the default settings provided by DARTS were applied. For N-HiTS, an architecture of three stacks and a single block in each stack was selected. Each block was composed of two dense layers with 512 neurons followed by ReLU activation. Finally, for TCN we used a kernel size of 4, a total of 64 filters, and a default base dilation of 2. For the training settings, a window size of 144 was used, with 200 epochs, a batch size of 256, and a learning rate of $10^{-3}$, with Adam as the optimizer and a dropout probability of 0.1. Again, as most of these settings were the default ones provided by DARTS, no hyperparameter tuning was conducted.

### 2.5. Model Evaluation

We evaluated the performance of each model using four different metrics: Mean Absolute Error (MAE), Mean Absolute Percentage Error (MAPE), Mean Squared Error (MSE), and Symmetric Mean Absolute Percentage Error (SMAPE). Given two time series, namely, *y* (real) and $\hat{y}$ (predicted), with both composed of *N* observations, the metrics are defined as follows:

(1) $$\mathrm{MAPE}\left(y,\hat{y}\right)\;=\;\frac{100}{N}\sum_{i=1}^{N}\left|\frac{y_{i}-{\hat{y}}_{i}}{y_{i}}\right|,$$

(2) $$\mathrm{MAE}\left(y,\hat{y}\right)=\frac{1}{N}\sum_{i=1}^{N}\left|y_{i}-{\hat{y}}_{i}\right|,$$

(3) $$\mathrm{MSE}\left(y,\hat{y}\right)=\frac{1}{N}\sum_{i=1}^{N}{\left(y_{i}-{\hat{y}}_{i}\right)}^{2},$$

(4) $$\mathrm{SMAPE}\left(y,\hat{y}\right)\;=\frac{100}{N}\sum_{i=1}^{N}\frac{|y_{i}-{\hat{y}}_{i}|}{\left(|{\hat{y}}_{i}\left|+\right|y_{i}|\right)/2}.$$

However, to calculate the overall performance of a model, we aggregated the performance obtained in each of the F=8 folds and each of the S=15 schools. Then, given a metric P∈{MAE,MAPE,MSE,SMAPE}, a model *m* from the models in Section 2.4, and a fixed prediction horizon *t*, the overall performance of *m* is provided by

(5) $$OvP\left(m,t\right)=\frac{1}{S}\sum_{s=1}^{S}\left(\frac{1}{F}\sum_{f=1}^{F}P_{\left(f,s\right)}\left(y,\hat{y}\right)\right).$$

Because this study analyses several prediction horizons, the following values are considered: t=1 (CO_2_ level in 10 min), t=3 (CO_2_ level in 30 min), t=6 (CO_2_ level in 1 h), t=9 (CO_2_ level in 1 h and 30 min), t=12 (CO_2_ level in 2 h), and t=24 (CO_2_ level in 4 h).

### 2.6. Implementation Tools

Training and processing of the models used in this study was performed using a Graphics Processing Unit (GPU) on a device equipped with a 2.9 GHz AMD Ryzen 7 4800H processor (AMD, Santa Clara, CA, USA). An NVIDIA GeForce GTX 1650 (NVIDIA Corporation, Santa Clara, CA, USA) graphics card with 12 GB of RAM was used. The version of Python we used was 3.7. For TS analysis and modeling, we utilized the open-source DARTS (Data Analytics and Reporting Toolkit for Time Series) library [39] (version 0.27.2) developed by Unit8. DARTS offers a comprehensive suite of functionalities specifically tailored for TS data manipulation and modeling.

Training DL models with GPUs often involves inherent probabilistic algorithms, regardless of seed fixation. This introduces randomness into the N-HiTS model, which can influence its results. To mitigate this and ensure unbiased results, the entire experimental process with N-HiTS was executed three times. The overall performance across different prediction horizons (evaluated by Equation (5)) was then calculated for each run. The average of the global metrics across the three runs is reported as the final performance of the N-HiTS model.

## 3. Results and Discussion

After running the entire experimental process in all fifteen schools ($S_{1}$ to $S_{15}$), the performance metrics (MAPE, MAE, MSE, SMAPE) of the proposed models (SH, MA, ARIMA, RF, XGB, N-HiTS, TCN) were obtained for the different evaluated prediction horizons (t=1, t=3, t=4, t=5, t=12, t=24) using Equation (5). Table 3 presents the final results, with the best performing model highlighted for each prediction horizon and metric.

As shown in Table 3, SH achieves the highest performance in most metrics for the shortest prediction horizon (t=1), except for MSE, where TCN performs best. For t=3, TCN outperforms all other models in every metric. This pattern is consistent for horizons t=6, t=9, and t=12, with TCN showing the best results in all metrics. At the longest horizon (t=24), N-HiTS achieves the best values in terms of MAPE, MAE, and SMAPE, while TCN continues to perform best in terms of MSE.

These results highlight the effectiveness of simple models such as SH for very short-term predictions (t=1). However, the accuracy of SH deteriorates as the forecasting horizon extends. ML-based models such as TCN begin to outperform simpler models as early as t=3, with this trend becoming increasingly pronounced at longer horizons. Classic ML models such as RF and XGB, while unable to outperform SH or ARIMA at shorter horizons, start performing better from t=9 onward (RF), following a pattern similar to their DL counterpart (TCN). In contrast, the N-HiTS model exhibits weaker performance compared to simpler ML models such as RF up to the longest horizon (t=24). Specifically, N-HiTS does not outperform SH in terms of MAPE until t=12, demonstrating that it has difficulty handling shorter and medium-term predictions effectively. ARIMA, with a similar behavior to SH (although always underperforming it), shows relatively stable performance for shorter horizons, although its accuracy declines as the prediction horizon increases.

**Figure 5:**
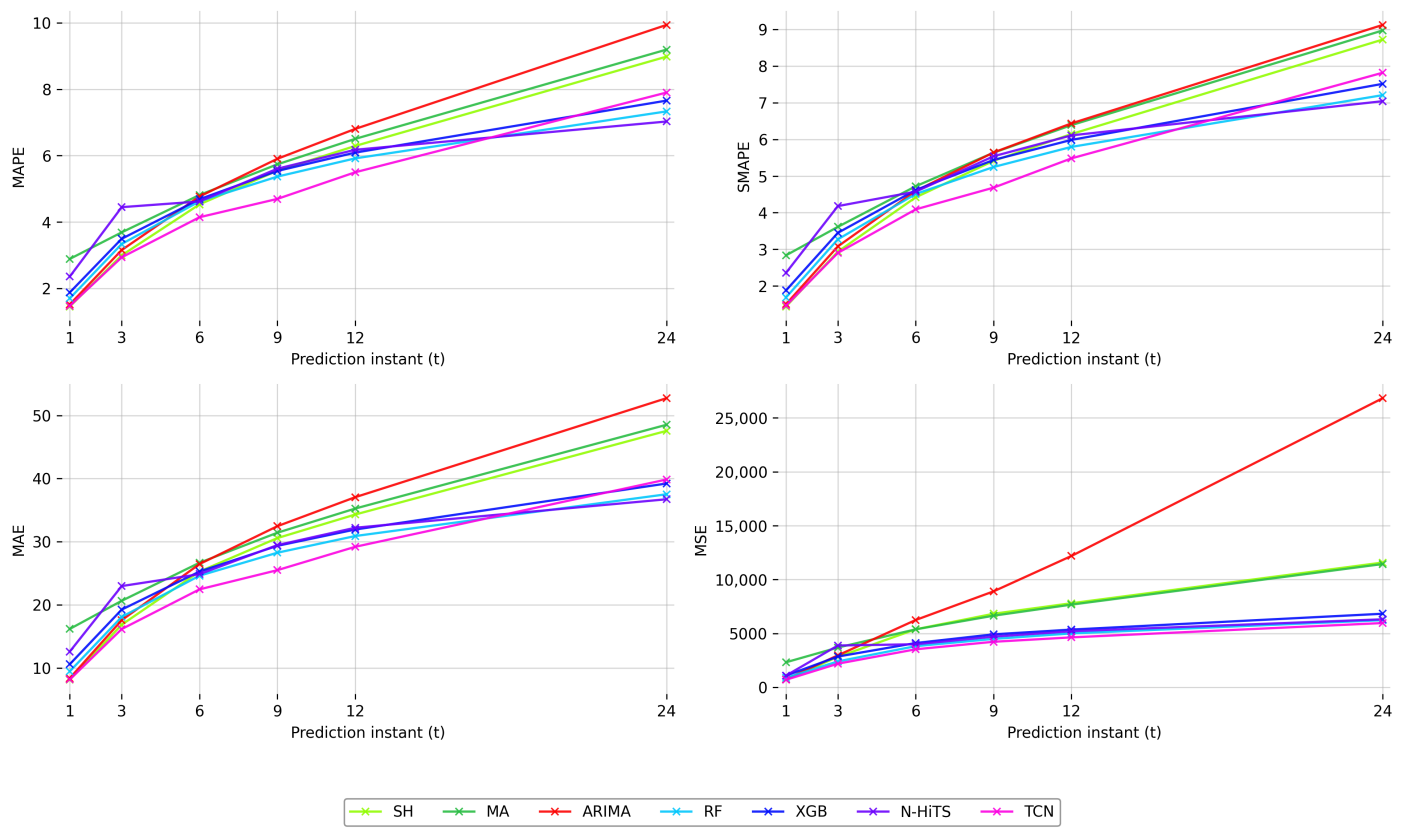
Evolution of model performance at different forecast times.

Overall, the results show how performance varies with prediction horizons; simple models such as SH and ARIMA perform well in very short-term predictions, while ML-based approaches, particularly TCN, excel in medium and long-term horizons. Figure 5 further illustrates this trend, showing the evolution of the different performance metrics across all horizons. As the prediction horizon increases, the results of all models naturally decline due to the growing complexity associated with forecasting further into the future. However, the ML models exhibit a smoother decline in performance compared to the simpler models, as seen in Figure 5, allowing them to generalize better for extended prediction horizons. It should be noted that a larger prediction horizon also provides earlier estimates of CO_2_ levels, facilitating informed decision-making to address the impact of this pollutant.

**Figure 6:**
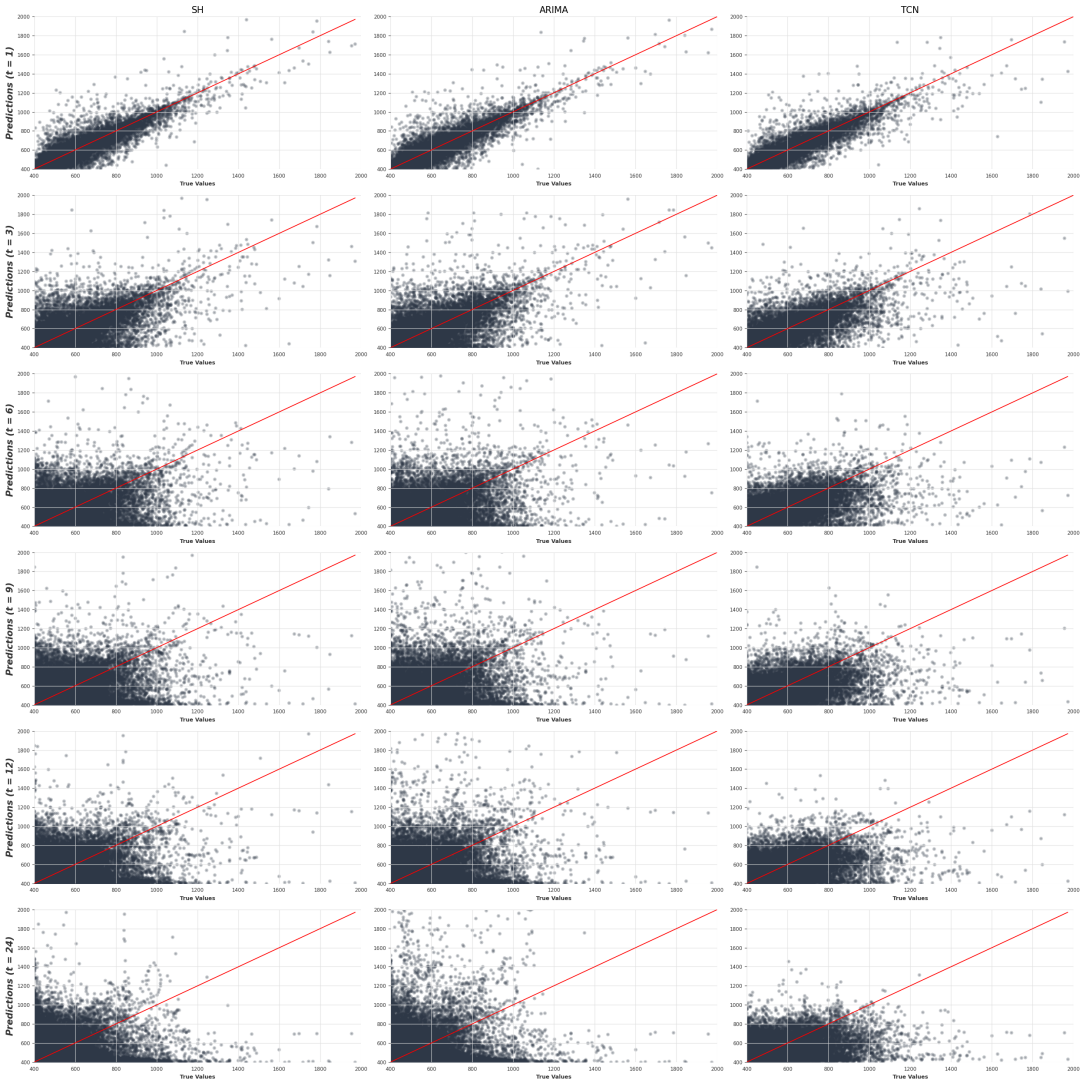
Relationship between the observed CO_2_ levels and those predicted by the SH, ARIMA, and TCN models in the different evaluated forecast horizons.

Finally, Figure 6 presents scatterplot density visualizations across different prediction horizons (vertical axis) for the selected representative models of each forecasting strategy, namely, SH, ARIMA, and TCN (horizontal axis). These plots compare the true CO_2_ values with the predicted values, with the red diagonal line representing an ideal predictor. While prediction dispersion increases with longer horizons, indicating a decline in accuracy, the TCN model demonstrates a more consistent distribution compared to SH and ARIMA. This observation is consistent with the performance metrics reported in Table 3, highlighting the ability of the DL models to maintain greater stability in predictions over extended time periods.

### Statistical Test

Having identified the best-performing model for each prediction horizon, we are now interested in determining whether there are any statistical differences among models by applying nonparametric statistical tests. Specifically, the Aligned-Friedman rank test [40] was used to detect statistical differences between the performance of the models in the prediction instants. The Holm post hoc test [41] was then applied to identify which models performed significantly different from the best model at each prediction horizon. Such tests are proposed in the studies [42,43,44,45], which suggest that their use in the ML area is highly recommended. The complete description of the tests and the software for their use can be found at http://sci2s.ugr.es/sicidm/, (accessed on 23 January 2025). The adjusted *p*-value (APV) was calculated to account for multiple comparisons, allowing for direct comparison with the chosen significance level (α=0.05) to reject the null hypothesis. The statistical tests were conducted considering the performance of each model and prediction horizon in each of the fifteen schools. In this study, all statistical tests were performed using MAPE as the primary performance metric.

The results obtained after applying the tests are summarized in Table 4 and Table 5. Examining the *p*-values from the Aligned-Frieman test (see Table 4), statistical evidence indicates that the models differ significantly in terms of performance, with the best model highlighted in the table.

The Holm post hoc test results in Table 5 reveal that SH is statistically superior to MA, XGB, and N-HiTS at t=1, with no significant difference observed between SH and ARIMA, RF, or TCN. At t=3, TCN emerges as the best model, showing statistically significant superiority over MA, XGB, and N-HiTS, although not over SH, ARIMA, or RF. By t=6 and t=9, TCN is statistically better than all other models. At t=12, TCN remains superior to all models except RF. Finally, at t=24, N-HiTS outperforms the non-ML-based models, although it does not show significant differences with RF, XGB, or TCN.

From the results obtained in the statistical tests and shown in Table 6, TCN stands out as one of the best performing methods over all prediction horizons. This robustness in various prediction scenarios positions TCN as a strong candidate for use as the default model in similar prediction tasks.

## 4. Conclusions and Future Work

This study investigated CO_2_ forecasting in school environments using data collected from IoT devices in fifteen schools between January and April 2022. Models representing various methodologies were trained using the data, then their performance was evaluated across different prediction horizons.

From the study, it was observed that simple models performed well for short-term CO_2_ prediction without requiring high computational costs. For instance, the SH model achieved better MAPE for very short-term forecasts (10 min), although it was not statistically superior to the other models.

Conversely, the ML models exhibited stronger performance as the prediction horizon was extended and the problem complexity increased. Notably, the TCN model achieved statistically significant improvements in predictive accuracy at intermediate horizons of t=6 and t=9 (1 h and 1.5 h), while the N-HiTS model demonstrated very good performance at the longest prediction horizon t=24 (4 h).

Although the ML models demonstrably outperformed simpler approaches for long-term predictions, this study found no statistically significant advantage for DL models such as N-HiTS over classical ML models such as RF and XGB or other DL models such as TCN. It should be noted that the DL architectures in this study were not exhaustively optimized, and we believe that further exploration and customization of DL architectures has the potential to yield superior performance, as we point out below in our discussion of future research lines.

In conclusion, this study proposes an effective and economical alternative to solutions that require structural modifications such as intelligent ventilation systems or devices to detect window opening and occupancy. Our results indicate that forecasting techniques represent a promising line of research for CO_2_ prediction in school classrooms, particularly those using DL models.

The novelty of this study lies in presenting a methodological framework for CO_2_ forecasting using data from low-cost IoT sensors. It combines robust validation, statistical analysis, and scale-independent metrics across multiple prediction horizons, thereby serving as a reference for future IAQ studies integrating low-cost monitoring and predictive modeling.

As future research directions, it is essential to investigate how other environmental parameters measured by MICA devices in addition to CO_2_ may influence forecasting models and contribute to improving prediction accuracy. Addressing this challenge requires a comprehensive multivariate approach capable of capturing not only linear interactions but also complex nonlinear dependencies and potential causal relationships. Furthermore, external factors such as building characteristics, occupant behavior, and ventilation strategies, which are known to affect IAQ and CO_2_ dynamics, must be considered as well.

Furthermore, collecting real-world data in uncontrolled environments such as schools by using low-cost sensors over extended periods of time is challenging. To address this limitation, we are actively participating in ongoing European projects that involve data collection in more controlled environments such as hospitals, nursing homes, subway stations, offices, and commercial spaces. These initiatives aim to obtain larger datasets over longer periods, which can enhance the reliability of future predictive models.

Additionally, the conventional loss functions commonly used in model training are often insufficient for managing the extreme values typical of CO_2_ distributions in classrooms during periods of occupancy, potentially hindering model performance. Addressing this limitation could significantly improve model accuracy, particularly for predictions of pollutants and other time series characterized by extreme value distributions. This topic has already been explored in some previous studies [46].

Finally, as no optimization tasks were performed on the models used in this study, we believe that analyzing hyperparameters or exploring new architectures could improve upon our results and further enhance predictive accuracy, particularly for long-term forecasting horizons.

## Figures and Tables

**Table 1:** Summary statistics of CO_2_ concentrations (ppm) measured in the classrooms of the fifteen schools S_1_–S_15_ used in the study (10 January 2022 to 3 April 2022) during occupied and unoccupied hours, showing the overall global results.

School	Non-Occupation			Occupation			Global	
	Mean	Std	Max	Mean	Std	Max	Mean	Std
$S_{1}$	408.31	9.46	438.00	432.54	49.57	849.00	430.26	47.79
$S_{2}$	409.30	11.29	447.00	462.74	92.42	1016.00	457.72	89.41
$S_{3}$	405.23	7.69	436.00	450.12	101.97	1262.00	445.90	97.97
$S_{4}$	401.81	3.66	419.00	431.77	59.91	924.00	428.95	57.70
$S_{5}$	404.82	7.37	429.00	497.35	139.44	1954.00	488.65	135.46
$S_{6}$	404.70	7.54	446.00	455.03	86.88	1137.00	450.30	84.02
$S_{7}$	409.86	9.62	445.00	453.20	122.30	1971.00	449.12	117.12
$S_{8}$	410.30	11.37	463.00	457.38	115.82	1599.00	452.93	111.12
$S_{9}$	404.38	6.85	443.00	431.34	63.54	1081.00	428.81	61.02
$S_{10}$	405.15	6.94	444.00	436.79	49.99	734.00	433.81	48.51
$S_{11}$	417.11	24.84	515.00	478.23	118.44	1639.00	472.48	114.39
$S_{12}$	401.53	3.79	425.00	441.62	55.82	977.00	437.84	54.41
$S_{13}$	403.92	6.83	431.00	468.97	119.93	1562.00	462.84	115.73
$S_{14}$	409.35	10.19	446.00	458.75	109.62	1125.00	454.11	105.37
$S_{15}$	404.44	6.61	434.00	432.71	45.17	827.00	430.05	43.82
AVG	406.68	8.94	444.07	452.57	88.72	1243.80	448.25	85.59

**Table 2:** Distribution of periods for the start and end dates of the training and validation sets for each fold.

	TRAIN		VAL	
#Fold	*Start-Date*	*End-Date*	*Start-Date*	*End-Date*
1	10-Jan-2022	6-Feb-2022	7-Feb-2022	13-Feb-2022
2	17-Jan-2022	13-Feb-2022	14-Feb-2022	20-Feb-2022
3	24-Jan-2022	20-Feb-2022	21-Feb-2022	27-Feb-2022
4	31-Jan-2022	27-Feb-2022	28-Feb-2022	6-Mar-2022
5	7-Feb-2022	6-Mar-2022	7-Mar-2022	13-Mar-2022
6	14-Feb-2022	13-Mar-2022	14-Mar-2022	20-Mar-2022
7	21-Feb-2022	20-Mar-2022	21-Mar-2022	27-Mar-2022
8	28-Feb-2022	27-Mar-2022	28-Mar-2022	3-Apr-2022

**Table 3:** Final results of the metrics obtained by each model in the different evaluated prediction horizons (*t*). In bold, the best result for each prediction instant and metric.

*Prediction Instant (t)*	Metric	SH	MA	ARIMA	RF	XGB	N-HiTS	TCN
1	MAPE	**1.452**	2.887	1.518	1.700	1.877	2.357	1.473
	MAE	**8.126**	16.183	8.389	9.472	10.595	12.599	8.176
	MSE	730.4	2319.3	750.8	871.6	1082.9	1083.7	**693.6**
	SMAPE	**1.441**	2.837	1.504	1.690	1.880	2.359	1.465
3	MAPE	2.995	3.688	3.154	3.337	3.494	4.447	**2.935**
	MAE	16.851	20.638	17.569	18.088	19.245	22.971	**16.160**
	MSE	2820.0	3685.6	2961.9	2402.8	2850.8	3875.4	**2189.2**
	SMAPE	2.937	3.614	3.081	3.274	3.451	4.180	**2.903**
6	MAPE	4.532	4.814	4.769	4.624	4.687	4.619	**4.144**
	MAE	25.276	26.666	26.458	24.616	25.272	24.858	**22.452**
	MSE	5378.7	5376.7	6242.7	3815.0	4104.4	3991.2	**3524.2**
	SMAPE	4.419	4.721	4.594	4.510	4.618	4.574	**4.091**
9	MAPE	5.546	5.738	5.905	5.368	5.539	5.607	**4.696**
	MAE	30.570	31.420	32.454	28.254	29.345	29.458	**25.490**
	MSE	6822.2	6641.8	8900.1	4488.3	4910.8	4698.9	**4221.5**
	SMAPE	5.414	5.636	5.642	5.246	5.438	5.538	**4.683**
12	MAPE	6.292	6.506	6.806	5.918	6.092	6.177	**5.499**
	MAE	34.301	35.248	37.047	30.895	31.929	32.208	**29.190**
	MSE	7797.5	7669.0	12183.4	4997.6	5351.3	5199.1	**4629.9**
	SMAPE	6.144	6.388	6.435	5.794	5.985	6.108	**5.481**
24	MAPE	8.985	9.197	9.941	7.334	7.659	**7.029**	7.902
	MAE	47.554	48.519	52.729	37.513	39.215	**36.719**	39.848
	MSE	11574.3	11436.8	26829.3	6226.0	6824.1	6307.2	**5960.7**
	SMAPE	8.721	8.970	9.121	7.206	7.514	**7.041**	7.817

**Table 4:** Results after applying the Aligned-Friedman test in predictions with different horizons (*t*). The F_*r*_ statistic and *p*-value of the test are shown along with the AFR (Aligned-Friedman Rank Average) ranks for each model. The F_*r*_ statistic is distributed according to chi-square with 6 degrees of freedom. For each prediction instant, the best test result (lowest value) is highlighted in bold.

*Prediction Instant (t)*	F_*r*_	*p*-Value	SH	MA	ARIMA	RF	XGB	N-HiTS	TCN
1	12.748	0.047	**23.8**	95.467	29.8	48.333	64.267	84.933	24.4
3	12.742	0.047	29.4	75.2	40.067	52.2	64.4	83.333	**26.4**
6	13.411	0.037	48.667	68.733	63.133	52.867	58.867	54.2	**24.533**
9	13.330	0.038	58.0	66.6	71.133	44.467	53.333	61.0	**16.467**
12	13.298	0.039	59.933	70.4	76.933	38.933	48.4	53.933	**22.467**
24	13.100	0.041	71.133	77.133	88.733	28.933	37.667	**22.333**	28.933

**Table 5:** APV at each prediction time (*t*) for each pair of models as a result of applying the Holm’s post hoc test together with the Aligned-Frieman test. The best model is selected as the model with the lowest rank (see Table 4).

*Prediction Instant (t)*	Best Model	SH	MA	ARIMA	RF	XGB	N-HiTS	TCN
1	SH	-	$6.958\times 10^{-10}$	1.179	0.082	0.001	$1.928\times 10^{-7}$	1.179
3	TCN	0.787	$5.713\times 10^{-5}$	0.438	0.061	0.003	$1.837\times 10^{-6}$	-
6	TCN	0.030	$4.229\times 10^{-4}$	0.003	0.023	0.008	0.023	-
9	TCN	$5.635\times 10^{-4}$	$3.269\times 10^{-5}$	$5.305\times 10^{-6}$	0.012	0.002	$2.485\times 10^{-4}$	-
12	TCN	0.003	$8.150\times 10^{-5}$	$5.814\times 10^{-6}$	0.139	0.039	0.014	-
24	N-HiTS	$4.570\times 10^{-5}$	$4.158\times 10^{-6}$	$1.415\times 10^{-8}$	0.553	0.336	-	0.123

**Table 6:** Summary table showing the best models at each time instant (*t*) resulting from the statistical tests. The best methods include the best performing method (in bold) as well as models that do not show statistically significant differences from the best model.

*Prediction Instant (t)*	Best Methods
1	**SH**, ARIMA, RF, TCN
3	SH, ARIMA, RF, **TCN**
6	**TCN**
9	**TCN**
12	RF, **TCN**
24	RF, XGB, **N-HiTS**, TCN

## Data Availability

Restrictions apply to the datasets. The datasets presented in this article are not readily available because the data are part of an ongoing study. Requests to access the datasets should be directed to P.G.-P.
